# Performance, Acceptability, and Usability of Respiratory Rate Timers and Pulse Oximeters When Used by Frontline Health Workers to Detect Symptoms of Pneumonia in Sub-Saharan Africa and Southeast Asia: Protocol for a Two-Phase, Multisite, Mixed-Methods Trial

**DOI:** 10.2196/10191

**Published:** 2018-10-25

**Authors:** Kevin Baker, Mucunguzi Akasiima, Alexandra Wharton-Smith, Tedila Habte, Lena Matata, Diana Nanyumba, Morris Okwir, Anteneh Sebsibe, Madeleine Marasciulo, Max Petzold, Karin Källander

**Affiliations:** 1 Department of Public Health Sciences Karolinska Institute Stockholm Sweden; 2 Malaria Consortium London United Kingdom; 3 Malaria Consortium Kampala Uganda; 4 Malaria Consortium Phnom Penh Cambodia; 5 Malaria Consortium Addis Ababa Ethiopia; 6 Malaria Consortium Juba Sudan; 7 Malaria Consortium Raleigh, NC United States; 8 Gothenburg University Gothenburg Sweden

**Keywords:** childhood pneumonia, diagnostic tools, low-income country, pulse oximeter, research design, respiratory rate counting

## Abstract

**Background:**

Pneumonia is one of the leading causes of death in children aged under 5 years in both sub-Saharan Africa and Southeast Asia. The current diagnostic criterion for pneumonia is based on the increased respiratory rate (RR) in children with cough or difficulty breathing. Low oxygen saturation, measured using pulse oximeters, is indicative of severe pneumonia. Health workers often find it difficult to accurately count the number of breaths, and the current RR counting devices are often difficult to use or unavailable. Nonetheless, improved counting devices and low-cost pulse oximeters are now available on the market.

**Objective:**

The objective of our study was to identify the most accurate, usable, and acceptable devices for the diagnosis of pneumonia symptoms by community health workers and first-level health facility workers or frontline health workers in resource-poor settings.

**Methods:**

This was a multicenter, prospective, two-stage, observational study to assess the performance and usability or acceptability of 9 potential diagnostic devices when used to detect symptoms of pneumonia in the hands of frontline health workers. Notably, 188 possible devices were ranked and scored, tested for suitability in a laboratory, and 5 pulse oximeters and 4 RR timers were evaluated for usability and performance by frontline health workers in hospital, health facility, and community settings. The performance was evaluated against 2 references over 3 months in Cambodia, Ethiopia, South Sudan, and Uganda. Furthermore, acceptability and usability was subsequently evaluated using both qualitative and quantitative methodologies in routine practice, over 3 months, in the 4 countries.

**Results:**

This project was funded in 2014, and data collection has been completed. Data analysis is currently under way, and the first results are expected to be submitted for publication in 2018.

**Conclusions:**

This is the first large-scale evaluation of tools to detect symptoms of pneumonia at the community level. In addition, selecting an appropriate reference standard against which the devices were measured was challenging given the lack of existing standards and differences of opinions among experts. The findings from this study will help create a standardized and validated protocol for future studies and support further comparative testing of diagnostic devices in these settings.

**Trial Registration:**

Australian New Zealand Clinical Trials Registry ACTRN12615000348550; https://www.anzctr.org.au/Trial/Registration/TrialReview.aspx?id=367306&isReview=true (Archived by Website at http://www.webcitation.org/72OcvgBcf)

**International Registered Report Identifier (IRRID):**

RR1-10.2196/10191

## Introduction

### Background

Pneumonia is one of the leading causes of death in children aged under 5 years, accounting for an annual 944,000 deaths globally, and 60% of these deaths occur in just 10 countries in South Asia and sub-Saharan Africa [1]. Deaths from pneumonia in children result mostly from delayed presentation to appropriate care, inappropriate treatment, or presuming the symptoms are of malaria [1]. Children with severe pneumonia have additional symptoms and danger signs such as chest in-drawing, stridor, and wheezing, which some health care workers are not able to adequately recognize, and subsequently, such children are treated with or referred for antibiotic treatment and oxygen therapy [2].

To diagnose pneumonia, frontline health workers are taught to observe a child’s chest for a full minute to visually identify and count the child’s breaths or respiratory rate (RR) and assess whether the RR is higher than the normal parameters for a child of that age, as defined by the World Health Organization (WHO) [3]. Counting the RR can be challenging in itself for health workers, and the misclassification of observed rate is common.

However, even with the deployment of the 1-minute acute respiratory infection timer in the early 1990s, counting the RR continues to prove challenging, as children breathe irregularly and faster than adults and may not sit still for a full minute. Misclassification of the observed rate is, therefore, still common, leading to incorrect diagnosis and, consequently, inappropriate treatment [4-7].

In addition, the acute respiratory infection timer has several shortcomings, such as short battery life and a distracting ticking sound every second, which can lead a health worker to count the sound instead of the chest movements. In a recent observational study, only 3 of 10 Mozambican community health workers (CHWs) counted the RR in children with a cough; of them, 1 counted the ticking of the timer, resulting in an RR of 60 breaths per minute, whereas other CHWs carried the timer but never used it, and the timer did not work in one case [8].

In addition, delays in seeking treatment put children at risk of developing severe pneumonia, and the inability of health care workers to adequately recognize danger signs and urgently refer children to a higher level of care leads to the death of many children. Hypoxemia, a symptom of severe pneumonia, has been identified as a predictor for morbidity and mortality in children with respiratory illness [9]. However, hypoxemia is poorly identified on the basis of clinical findings alone [10]. While pulse oximetry can be used to measure oxygen saturation (SpO_2_) and is a reliable and noninvasive method for identifying children with hypoxemia, pulse oximeters are rarely available outside of higher-level facilities in resource-constrained countries.

Integrated community case management (iCCM) is an approach developed by the WHO, United Nations Children’s Fund (UNICEF), and partners [11] where CHWs are trained to identify and treat pneumonia, malaria, diarrhea, and malnutrition in children aged under 5 years, as well as refer severely ill children to the nearest health facility. Evidence in African countries shows that health workers, if properly trained and equipped, can potentially reduce child deaths from malaria, pneumonia, and diarrhea by up to 60% through the delivery of iCCM [12]. The integrated management of childhood illness (IMCI) was developed by the WHO to support health workers in health centers to better manage childhood illnesses [13].

More recently, and partly as a response to the scale-up of large iCCM projects in sub-Saharan Africa and Southeast Asia, new pneumonia diagnostic aids have been developed by industry, academia, and other partners to improve the accuracy and effectiveness of diagnosing pneumonia in resource-poor contexts [14].

### Study Aim and Objectives

This paper presents the protocol of a study aimed to identify the most accurate, acceptable, scalable, and user-friendly RR counters and pulse oximeters for the diagnosis of pneumonia symptoms in children by CHWs and first-level health facility workers (FLHFWs) with different levels of training in 4 countries: Cambodia, Ethiopia, South Sudan, and Uganda. The two main objectives were (1) to evaluate the accuracy of 9 RR counters and pulse oximeters in the hands of CHWs and FLHFWs in 4 low-resource countries and (2) to assess the usability and acceptability of the 9 devices among CHWs and FLHFWs in 4 low-resource countries when used in the routine practice over a 3-month period.

## Methods

### Ethical Approval and Consent to Participate

The study was approved by the ethical review boards in each study country at the national or regional level: in Ethiopia, from the Southern Nations Nationalities Peoples’ Region Health Bureau Health Research Review Committee (Ref: 6-19/10342); in Uganda, from the Uganda National Council for Science and Technology (Ref: HS 1585); in South Sudan, from the Research and Ethics Committee at the Government of South Sudan, Ministry of Health (dated May 23, 2014); in Cambodia, from the National Ethics Committee for Health Research (Ref: 0146 NECHR); and by the Regional Ethics Committee in Stockholm, Sweden (Ref. 2017/4:10). Participants were recruited only after obtaining written informed consent. The clinical evaluation is registered with the Australia New Zealand Clinical Trials Registry (ACTRN12615000348550).

### Study Design

**Figure 1:**
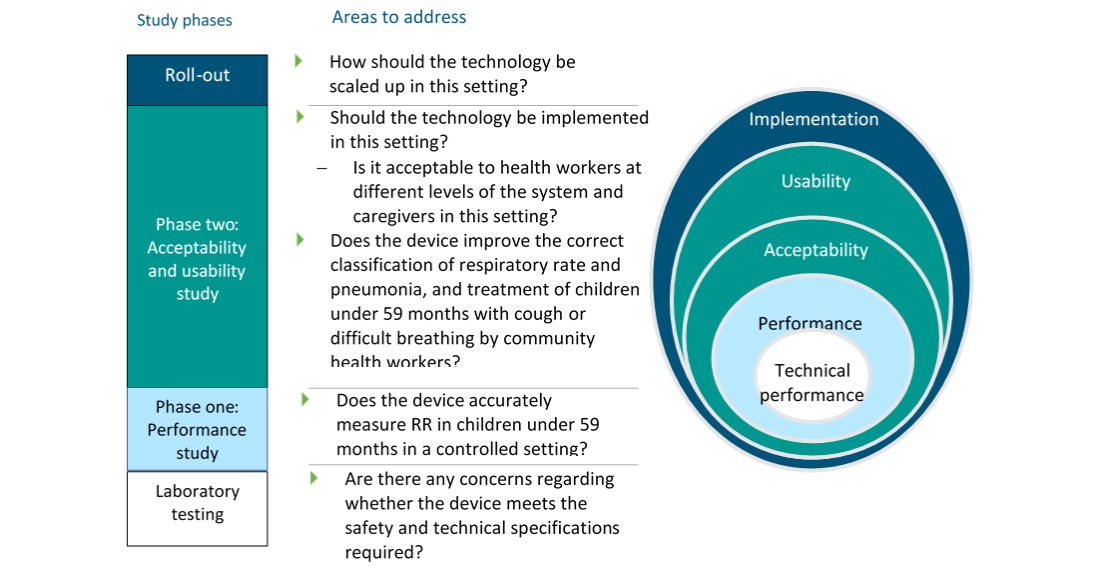
Stages of introducing a new technology.

A two-phase, mixed-methods design was developed to examine the performance, acceptability, and usability of the 9 devices in the 4 countries (Figure 1). The conceptual framework for the design was adapted from the WHO documents “Health technology assessment of medical devices” [15] and “Introducing new technology safely” [16].

The first phase, the performance evaluation element of the study, was a multicenter, single-blind comparison of the performance of devices to detect symptoms of pneumonia in the hands of frontline health workers using 2 reference standards. The second phase, the acceptability and usability evaluation element of the study, was a mixed-methods, multicenter, observational study using both qualitative and quantitative data to compare the acceptability and usability of devices to detect symptoms of pneumonia in the hands of CHWs and FLHFWs in routine practice.

### Study Sites

This was a multicountry study implemented in Cambodia, Ethiopia, South Sudan, and Uganda as all 4 countries have a high proportion of deaths of children under 5 years caused by pneumonia (16%-21%), as well as a high incidence of pneumonia, and all have implemented the Ministry of Health-defined iCCM and IMCI programs. However, the characteristics of the health worker programs, such as the length of training, literacy level, and RR timing devices used, differed by country (Table 1).

The study sites selected for conducting phase 1 were all district hospital-level facilities selected after the analysis was conducted on patient flow to understand whether the individual research sites could support the sample size required by the study for enrollment, as it would not have been possible to achieve the sample if phase 1 had been conducted at the community level (Table 2).

Phase 1 research sites were as follows: Mpigi General Hospital, approximately 45 miles from Kampala in Uganda; Yrgalem District Hospital in Southern Nations and Nationalities and People’s Region in Ethiopia; Borkeo District Hospital in Ratanakiri province in Cambodia; and Aweil General Hospital in Northern Bahr el Ghazal state in South Sudan. For phase 2 of the study, frontline health workers were selected depending on having participated in phase 1 and being within 20 km of the health facility used in phase 1 in order to have access to the functioning oxygen equipment and severe illness case management capabilities.

**Table 1:** Country implementation of pneumonia diagnosis and treatment at community level.

Characteristics	Cambodia	Ethiopia	South Sudan	Uganda
Pneumonia deaths (% of total under-5 deaths) [17]	17	17	21	16
Pneumonia incidence in children under 5 years (number of episodes per child per year) [18]	0.25	0.28	0.32^a^	0.27
Proportion of children aged under 5 years with suspected pneumonia and receiving antibiotics (%) [19]	39	7	33	47
Name for community health worker (CHW)	Extended village malaria worker	Health extension worker	Community drug distributor	Village health team member
Length of initial training	5 days (2 days malaria training + 3 days in sick child case management)	1 year	6 days	11 days (5 days basic training + 6 days in sick child case management)
Literacy level	Low	High	Extremely low	Low-median
Pneumonia diagnosis tool	Acute respiratory infection (ARI) timer	Wrist watch or ARI timer	ARI timer + beads	ARI timer
Catchment population per CHW	130-150 households	400-500 households	250-300 households	250-500 households
Average caseload per month	8	12	9	12

^a^Data are for Sudan.

**Table 2:** Demographic characteristics of the study sites in Cambodia, Ethiopia, South Sudan, and Uganda.

Description	Cambodia Ratanakiri province	Ethiopia (Dale & Shebedino Districts), SNNPR^a^	South Sudan (Aweil West & Center counties) Northern Bahr el Ghazal State	Uganda, Mpigi District
Population	184,000	529,041	128,295	250,548
Children under 5 years population, N (%)	37,720 (20.50)	82,582 (15.61)	25,000 (19.49)	51,363 (20.50)
Number of community health workers	270	161	1683	650
Number of health centers	23	19	20	39
Number of hospitals	2	2	1	1

^a^SNNPR: Southern Nations and Nationalities and People’s Region.

### Device Selection

A number of activities were conducted before the field trials to select the test devices. First, the formative research was conducted to inform the attributes used in the subsequent device scoring [20]. Second, an initial landscape review was conducted where 188 possible devices were identified [21]; these devices were further evaluated in a review of technical specifications [22]. Third, all potential devices were scored and ranked using 20 device attributes, including measures of usability, utility, scalability, and user acceptance. Multimedia Appendix 1 provides the table with the device scoring based on the attributes. In contrast to the respiratory timing devices identified, the 8 selected pulse oximeters had not previously been field-tested, and before taking them to the field trials, they were first tested in a laboratory for accuracy and environmental robustness. Based on the laboratory test results, the final 9 devices were taken forward to the performance evaluation phase (Multimedia Appendix 2), that is, 4 RR devices (manual and assisted counters) and 5 pulse oximeters (fingertip and handheld devices) [23].

The devices allocated to each country were based on the suitability for individual country context; overall, 9 devices were tested for performance (phase one) and the usability and acceptability in the routine practice (phase 2).

### Sample Size

The sample size calculation for phase 1 was based on the precision of the mean difference between the device and the reference respiratory count, assuming the normal distribution. An SD of 7 for the difference was obtained in a previous study evaluating the performance of RR counters [24] and in requiring a maximal total length of the 95% CI of 4 units, which is the same range as the WHO-accepted maximal absolute breathing rate deviance (eg, ±2 breaths per min); the minimum sample size was 47 children per strata for independent observations. The two age strata in the study were (1) 0-60 days and (2) 2-59 months, and 3 device pairs per country gave a total sample size of 282 children. The sample size was then increased by 50% to 423 and rounded off to 430 children per country to accommodate for potential clustering at the CHW level [25]. For the usability and acceptability of the routine practice assessment, a sample of 20 CHWs and 5 FLHFWs was recruited to do the assessments in each country over the 3 months.

### Outcomes

The primary outcome for phase 1 was the agreement between each health worker measurement, using the test device, and that of the reference standard, calculated as the proportion of 1-minute observations that were within ±2 breaths or ±2% SpO_2_ for each of the 9 devices. The secondary outcomes included the agreement in classification of the breath rate into (1) normal or fast breathing and oxygen saturation into (2) normal oxygen saturation (SpO_2_≥90%) or hypoxemia (SpO_2_<90%) and included agreement statistics appropriate for situations when no gold standard exists, such as positive percent agreement, negative percent agreement, and Cohen kappa statistic.

The primary outcome for phase 2 (the usability and acceptability evaluation) was acceptability, based on users’ perceptions of the different devices used. Secondary outcomes for phase 2 included the proportion of users who accurately followed the correct procedures and the caregivers’ perceptions of, interaction with, and reaction to the devices when used on children.

### Study Procedures

In phase 1, all children aged 0-5 years presenting at the health facilities where the study was conducted were screened for eligibility and invited to participate. All young infants aged 0-60 days were eligible, as were children aged 2-59 months with a cough and/or difficulty in breathing. Children with an illness of >2-week duration or exhibiting one or more of the IMCI danger signs (severe dehydration, agitation, inconsolable, neck stiffness, active convulsions or fits, unconscious or lethargic, not breastfeeding, and vomiting everything) as well as those with severe burns, with neutropenia, with a severe infectious disease, or ineligible as advised by the supervising clinician were excluded.

In the absence of a gold standard, 2 reference standards were used for phase 1 of this study: (1) an IMCI-trained expert clinician (EC) and whose RR counting skills were standardized to ±2 breaths per minute on 5 video recorded children and (2) an automated monitoring device (Masimo Root patient monitoring and connectivity platform with Phasein ISA CO_2_ capnography using nasal cannulas and Radical 7 pulse oximeter) [26] that provided a measurement for the same period. All health workers received 2 days of training prior to data collection, including a refresher module on iCCM as well as practice sessions on RR counting and using a new device. All health workers needed to receive a pass mark of ≥90% on a competency-based assessment before participating in data collection. On consenting to the studying and entering the research room, a child was positioned comfortably on a caregiver’s lap and calmed and attached to the Masimo reference device. For the RR counters, 2 assessments were performed by the health workers and recorded, along with the corresponding Masimo reference measurements, and within 5 minutes of the health worker measurements, the ECs took 2 RR measurements using a stopwatch. For pulse oximeter devices, the health workers took 2 SpO_2_ measurements, and the EC also took 2 SpO_2_ measurements using the same pulse oximeter the health worker had used, along with simultaneous Masimo SpO_2_ reference measurements. The health workers were asked to classify a child into fast or normal breathing using the WHO age-specific cut-offs for RR for RR devices or for severe or nonsevere pneumonia based on the SpO_2_ reading being <90% for pulse oximeters.

In phase 2, to assess the usability and acceptability of the devices in routine practice by health workers and explore their acceptability to caregivers, field testing was conducted over 3 months. This was a mixed-methods study incorporating structured observations, video recordings of procedures, and qualitative exit interviews with health workers and caregivers. During the activity, 100 health workers were trained for 2 days to use an RR counter and a pulse oximeter as part of their routine iCCM or IMCI activity. Each health worker had to pass a competency-based assessment before participating in the data collection. The research team scheduled visits with each health worker 3 times (once a month) during the evaluation, each time gathering a minimum of 5 assessments of each CHW or FLHFW. The health workers took the medical history of children as per the iCCM or IMCI guidelines, and if cough and/or difficulty breathing were recorded, the health workers used the RR device to count the number of breaths in 1 minute; a procedure that was repeated twice, and the highest reading was used for classification. The observed RR was used by the health workers to decide whether or not to provide treatment for pneumonia using the national treatment guidelines. If fast breathing was detected, the health workers assessed for hypoxemia using the pulse oximeter by taking two SpO_2_ readings and used the lowest reading for classification. All children with signs and symptoms of severe pneumonia and with SpO_2_ <90% were referred.

### Data Collection

Paper-based data collection tools were developed in local languages for both phases and collected data on screening, usability and performance, and adverse events. All data collection tools were developed in collaboration across the 4 research sites and were translated and pilot-tested in all locations before the start of the study. The tools for performance and usability included demographic information, child status, device measurement results, health worker classification of results, time taken, failures, and usability checklists. In addition, semistructured interview guides were developed for the exit interviews with caregivers and health workers (Multimedia Appendix 3). Video recordings and photographs of the health worker log books were captured as back-up to the paper forms submitted. All completed forms and log book photos were returned to the Malaria Consortium office for double data entry using EpiData version 3.1 (EpiData Association, Odense, Denmark) and filed. The qualitative data collection consisted of in-depth interviews with health workers at the end of data collection to capture their views on the usability and acceptability of the devices. Each data form had a unique identification code to link data from different forms in the database.

### Data Analysis

The analysis of the primary outcome for phase 1, the agreement between each health worker measurement using the test device and that of the reference standards, was done following a per-protocol approach for each RR and SpO_2_ measurement. Agreement was calculated as the proportion of observations that were within ±2 breaths or SpO_2_% for each of the 9 devices, respectively, in the per-protocol population (only including children who had a cough and difficulty breathing and no danger signs). The secondary outcomes for the performance evaluation, the agreement in classification of the RR or SpO_2_ obtained by health workers and the reference standards was measured using Cohen kappa statistic, positive percent agreement, and negative percent agreement of each device. For all of these secondary outcomes, the unit of analysis was the child rather than the device measurements. To illustrate the agreement between different devices, Bland-Altman plots [27] were produced to visualize the agreement in ratings of respiratory counts between reference standards and CHWs. The primary outcome for the usability and acceptability evaluation in phase 2 used qualitative analysis to establish the acceptability, based on users’ perceptions of the different devices used. Secondary outcomes for the acceptability and usability evaluation included the proportion of users who accurately followed the correct procedures and the caregivers’ perceptions of, interaction with, and reaction to the devices when used on children.

## Results

This project was funded in 2014. Data collection started on February 4, 2015 and was completed on December 31, 2015. Data analysis is currently under way and the first results are expected to be submitted for publication in 2018.

## Discussion

To reduce the number of child deaths from pneumonia in low-resource settings and to minimize unnecessary use of antibiotics, it is crucial to improve the diagnosis of pneumonia symptoms by frontline health workers [28]. This study aimed to meet this need by identifying possible improved pneumonia diagnostic devices and evaluated 9 of these in “real-life” health system contexts in 4 different countries in 2 continents. One of the key factors in designing a study like this is agreeing on the gold or reference standard to compare the devices to. The gold standard is the best single test (or a combination of tests) that is considered the current preferred method of diagnosing a particular disease [29]. Many possible gold standards have been suggested for RR timing devices in the literature, such as simultaneous counting by a clinical expert [5], real-time electronic monitoring, or retrospective review of video recordings by a panel of experts [7,30]. However, all these methods have limitations such as the inaccuracy of human counters [30] and the inconsistency of counting between humans and electronic monitoring devices [30]. On review, and through discussions with experts from the WHO, UNICEF, and academia in the scientific advisory committee for the study, it was decided that no suitable gold standard exists in this setting; therefore, it was agreed to use 2 imperfect reference standards—the automated Masimo Root patient monitoring and connectivity platform with Radical-7 pulse oximeter and capnograph using Phasein ISA CO_2_ module [26] and a simultaneous assessment by a clinical expert. Furthermore, it was hoped that using 2 different types of references, in the absence of a suitable gold standard, would best account for the limitations outlined above.

## Appendix

Multimedia Appendix 1 Complete scoring sheet for 188 possible pneumonia diagnostic aids, including respiratory rate timers, pulse oximeters and joint devices.

Multimedia Appendix 2 Summary of final devices tested in the field for performance, acceptability, and usability.

Multimedia Appendix 3 Discussion guide for caregiver interviews.

## Abbreviations

CHW: community health worker
EC: expert clinician
FLHFW: first-level health facility workers
iCCM: integrated community case management
IMCI: integrated management of childhood illness
RR: respiratory rate
UNICEF: United Nations Children’s Fund
WHO: World Health Organization
