# Metadata Correction: Performance, Acceptability, and Usability of Respiratory Rate Timers and Pulse Oximeters When Used by Frontline Health Workers to Detect Symptoms of Pneumonia in Sub-Saharan Africa and Southeast Asia: Protocol for a Two-Phase, Multisite, Mixed-Methods Trial

**DOI:** 10.2196/13755

**Published:** 2019-03-07

**Authors:** Kevin Baker, Mucunguzi Akasiima, Alexandra Wharton-Smith, Tedila Habte, Lena Matata, Diana Nanyumba, Morris Okwir, Anteneh Sebsibe, Madeleine Marasciulo, Max Petzold, Karin Källander

**Affiliations:** 1 Department of Public Health Sciences Karolinska Institute Stockholm Sweden; 2 Malaria Consortium London United Kingdom; 3 Malaria Consortium Kampala Uganda; 4 Malaria Consortium Phnom Penh Cambodia; 5 Malaria Consortium Addis Ababa Ethiopia; 6 Malaria Consortium Juba Sudan; 7 Malaria Consortium Raleigh, NC United States; 8 Gothenburg University Gothenburg Sweden

The authors of the paper “Performance, Acceptability, and Usability of Respiratory Rate Timers and Pulse Oximeters When Used by Frontline Health Workers to Detect Symptoms of Pneumonia in Sub-Saharan Africa and Southeast Asia: Protocol for a Two-Phase, Multisite, Mixed-Methods Trial” (JMIR Res Protoc 2018;7(10):e10191), made a mistake in the final stage of proofreading. Kevin Baker should have been listed as affiliated with the Karolinska Institute in Stockholm, Sweden. This affiliation has also been updated to include the Department of Public Health Sciences for all affiliated authors and now reads as follows:

Department of Public Health Sciences, Karolinska Institute, Stockholm, Sweden

Additionally, Kevin Baker's address as the corresponding author for this paper has been changed to reflect this new affiliation.

The correction will appear in the online version of the paper on the JMIR website on March 7, 2019, together with the publication of this correction notice. Because this was made after submission to PubMed, PubMed Central, and other full-text repositories, the corrected article also has been resubmitted to those repositories.

