# Supplementary material for: Performance, Acceptability, and Usability of Respiratory Rate Timers and Pulse Oximeters When Used by Frontline Health Workers to Detect Symptoms of Pneumonia in Sub-Saharan Africa and Southeast Asia: Protocol for a Two-Phase, Multisite, Mixed-Methods Trial
Source: JMIR Res Protoc. 2018 Oct 25;7(10):e10191. doi: 10.2196/10191 (PMC6231813; doi:10.2196/10191)
Supplement: Multimedia Appendix 2 [file resprot_v7i10e10191_app2.pdf]

**Mark 2 Acute Respiratory Infection timer (Model: MK2 ARI):** This manual counting device has a push button to start the timer, a flashing light to show it is counting and a beep sound at the end of 60 seconds. Battery operated, with a stated a lifespan of 2 years. The health worker uses this device to count 60 seconds while manually counting the respiratory rate of the child through observation of the child's chest area.	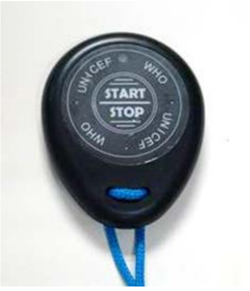
**Beads with ARI timer:** The beads are used in conjunction with the MK2 ARI timer to support the health worker to count respiratory rate by moving a bead along each time they see a chest movement. Health workers have three sets of beads, one for each respiratory rate cut-off age group, each containing 40, 50 or 60 beads of one colour (blue in the picture) and five beads of another colour (red in the picture) respectively. If the health worker finishes counting on a red coloured bead in the picture they should classify the child as having fast breathing pneumonia.	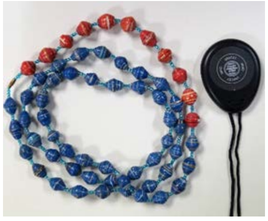
**Rrate smart phone application** is a smart phone tapping application that provides a respiratory rate count within a defined number of consistent breaths, which can be set from three to six, and a defined consistency threshold from 10 to 14%. The user taps the smart phone screen for each breath viewed. Once the application has provided a respiratory rate the user has to verify	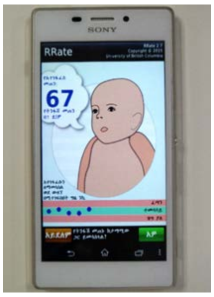

the consistency by watching and agreeing with the respiratory rate of an animated child on the screen, before confirming that the RR is correct.	
**Respirometer feature phone application** is a feature phone application that provides a respiratory rate reading after 10, 20 breath cycles and 60 seconds as well as categorisation of the RR (fast or normal) based on the age of the child. The user counts each breath by pressing a number button on the phone keypad. In this study the 60 second reading was used for all analysis.	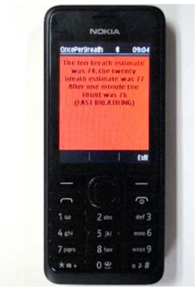
**Contec fingertip pulse oximeter (Model: CMS50QB)** measures oxygen saturation and pulse rate, through attaching the probe to the patient's finger or toe. This is a lower cost device supplied with two rechargeable batteries and recommended for use with paediatric patients due to its smaller size. This device is CE approved as a class IIb medical device.	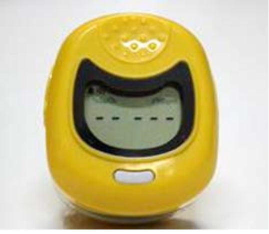
**Devon fingertip pulse oximeter (Model: PC600)** measures oxygen saturation and pulse rate, through attaching the probe to the patient's finger or toe. This is a higher cost device with a rechargeable battery recommended for use with paediatric patients. This device is CE approved as a class IIb medical device.	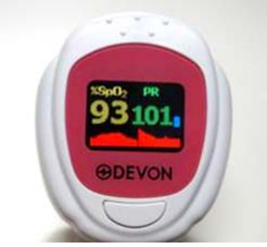

**Lifebox handheld pulse oximeter (Model: AH-M1)** is a robust compact hand held device measuring oxygen saturation and pulse rate with visual and audible alarms and comes with a warranty of two years. It is both battery and mains powered and is supplied with a rechargeable lithium battery. It can also be supplied with re-usable adult, neo-natal and paediatric probes. This device is CE approved as a class IIb medical device.	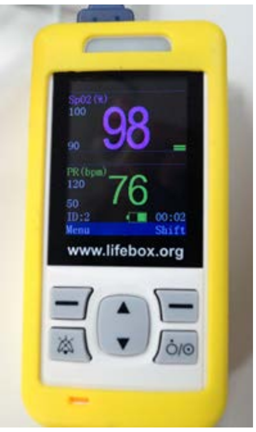
**Utech handheld pulse oximeter (Model: UT100)** is a compact, user-friendly and portable device, measuring oxygen saturation (SpO2) and pulse rate (PR) and can be used on adults, paediatric and neonatal patients. It is supplied with an adult reusable probe as standard and a one year warranty. While it can have re-chargeable batteries they need to be purchased additionally. This device is CE approved as a class IIb medical device.	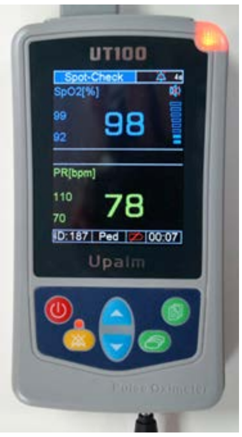
**Masimo mobile phone pulse oximeter (Model: iSpO2 Rx)** is a handheld pulse oximeter operating on a high specification android phone and is also available for iPhone. The device features low profusion and motion software supporting SpO2 assessments of adult, paediatric and neonatal patients with single and multi-use probes. This device is CE approved as a class IIb medical device	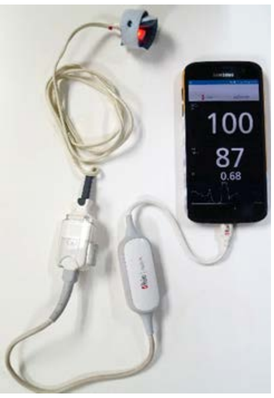
