# Supplementary material for: Performance, Acceptability, and Usability of Respiratory Rate Timers and Pulse Oximeters When Used by Frontline Health Workers to Detect Symptoms of Pneumonia in Sub-Saharan Africa and Southeast Asia: Protocol for a Two-Phase, Multisite, Mixed-Methods Trial
Source: JMIR Res Protoc. 2018 Oct 25;7(10):e10191. doi: 10.2196/10191 (PMC6231813; doi:10.2196/10191)
Supplement: Multimedia Appendix 3 [file resprot_v7i10e10191_app3.pdf]

malaria consortium
disease control, better health

Discussion Guide 2 (Stage 6): A study to capture caregivers perceptions and opinions of the tools used to diagnose pneumonia in their children.

ONE TO ONE INTERVIEWS

With

CAREGIVERS

INTERVIEWER: ___________________________________

COMMUNITY: _____________________________________

INTERVIEW NUMBER: _

DATE OF INTERVIEW (dd-mm-yy):

TIME STARTED (hh-mm):

TIME ENDED (hh-mm):

INTERVIEW participant UIC	Age	Sex

malaria consortium

disease control, better health

malaria consortium
disease control, better health

Introduction: Welcome and thank you so much for agreeing to be part of this research. Today we are going to discuss your recent experiences when you brought your child in to be diagnosed by your community health worker and your thoughts on the tools they used to diagnose pneumonia in your child.

1. How robust did you think this device was for the CHW to use?

2. How easy did this device look for the CHW to use?

3a. How accurate do you think the device is in diagnosing pneumonia? (Please circle)

Very Accurate	Fairly Accurate	Neither Accurate Nor Inaccurate	Fairly Inaccurate	Very Inaccurate

3b. Why?

4a. Was the device suitable to be used in your opinion? (Please circle)

Yes No

4b. Why?

5a. How well did your child react when the CHW used the device? (Please circle)

Very Well	Fairly Well	Neither well Nor Uneasy	Fairly Uneasy	Very Uneasy

5b. Why?

6a. Do you feel that this device helped the CHW to diagnose pneumonia? (Please circle)

Yes No

6b. Why?

7. Do you feel that this device made the CHWs job easier? (Please circle)

Yes No

8. Any other comments
